# EHMTI-0122. Serum micrornas as potential biomarkers of migraine

**DOI:** 10.1186/1129-2377-15-S1-F1

**Published:** 2014-09-18

**Authors:** HH Andersen, P Gazerani, M Duroux

**Affiliations:** 1 School of Health Science and Technology, Center of Sensory-Motor interaction Laboratory of Cancer Biology, Aalborg, Denmark; 2 School of Health Science and Technology, Center of Sensory-Motor interaction, Aalborg, Denmark; 3 School of Health Science and Technology, Laboratory of Cancer Biology, Aalborg, Denmark

## Background

Migraine is a highly prevalent neuro-vascular disorder. Protein-based serum biomarkers have been assessed in relation to diagnosis and patients' stratification, but general lack of robustness and reproducibility impede widespread clinical utilization. MicroRNAs have emerged as promising biomarkers for multiple conditions such as cancer and recently also for painful conditions.

## Aim

To assesses the potential of applying serum microRNAs as biomarkers for migraine.

## Methods

In an initial screening, 386 microRNAs, previously found in serum, were assessed in six migraineurs in pain-free periods and during migraine attacks. From this, four differentially expressed microRNAs (hsa-miR-34a-5p, miR-29c-5p, hsa-miR-382-5p and hsa-miR-26b-3p) were selected for further investigation. Validation was performed in a cohort of 8 migraineurs during attack, 8 in a pain-free period and 8 healthy sex- and age-matched controls and in a separate cohort of 12 migraineurs in pain-free period and 12 healthy age-matched controls. In total, serum microRNA expression was analyzed in 48 migraineurs and controls.

## Results

A significant alteration in more than 30 microRNAs (~8% of the assays) was detected during the screening. Three of the four microRNA were confirmed to have significantly higher expression during migraine attacks compared to pain-free periods. Furthermore, microRNA expression were significantly altered between migraineurs and healthy controls in two independent validation cohorts (n = 16 and n = 24).

## Conclusions

These preliminary data, proposes microRNAs as potential biomarkers for migraine with applications in patients' stratification, diagnosis or perhaps therapeutic aspects of migraine. Further investigations are warranted.

No conflict of interest.

